# A switch in N-terminal capping of β-peptides creates novel self-assembled nanoparticles†

**DOI:** 10.1039/d3ra04514e

**Published:** 2023-10-09

**Authors:** Yi-Kai Chen, Isabella A. Simon, Ivan Maslov, Ivan E. Oyarce-Pino, Ketav Kulkarni, Denham Hopper, Marie-Isabel Aguilar, Naveen Vankadari, Brad RS Broughton, Mark P. Del Borgo

**Affiliations:** a Department of Pharmacology, Monash University Clayton VIC 3800 Australia mark.delborgo@monash.edu; b Department of Biochemistry & Molecular Biology, Monash University Clayton VIC 3800 Australia; c Biomedicine Discovery Institute, Monash University Clayton VIC 3800 Australia; d Department of Biochemistry and Pharmacology, Bio21 Molecular Science and Biotechnology Institute, The University of Melbourne Melbourne VIC 3000 Australia

## Abstract

Small tripeptides composed entirely of β^3^-amino acids have been shown to self-assemble into fibres following acylation of the N-terminus. Given the use of Fmoc as a strategy to initiate self-assembly in α-peptides, we hypothesized that the acyl cap can be replaced by an Fmoc without perturbation to the self-assembly and enable simpler synthetic protocols. We therefore replaced the *N*-acyl cap for an Fmoc group and herein we show that these Fmoc-protected β^3^-peptides produce regular spherical particles, rather than fibrous structures, that are stable and capable of encapsulating cargo. We then demonstrated that these particles were able to deliver cargo to cells without any obvious signs of cytotoxicity. This is the first description of such regular nanoparticles derived from Fmoc-protected β^3^-peptides.

## Abstract 2

Alteration to the N-terminal cap of β-peptides switches self-assembly from fibrillar to spherical structures.

## Graphical abstract

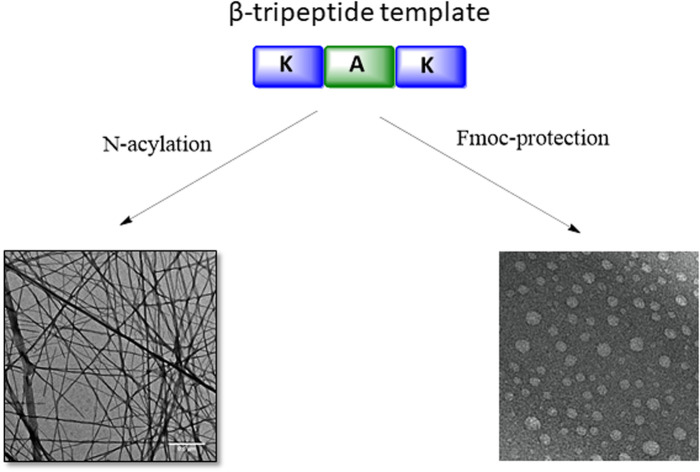

## Introduction

Peptides comprised entirely from β^3^-amino acids, otherwise known as β^3^-peptides have been shown to self-assemble into a range of fibrous structures following capping of the N-terminus.^1–5^ These β^3^-peptides self-assemble due to their unique structure, allowing the peptides to stack end-on-end.^2,6^ These structures can be tuned through the incorporation of lipid chains, which lead to the formation of injectable hydrogels.^7^ In addition, bioactive motifs can also be introduced to afford functional biomaterials.^8,9^

Previously, we have shown that the conjugation of 12, 14 or 16-carbon chains onto the N-terminus of β^3^-peptides results in homogenous self-assembly into fibrous structures.^8^ The addition of lipid chains onto the N-terminal is able to control self-assembly such that regular fibrillar structures are formed. This allows for the lipid chains and sequential β^3^-peptides to stack on top of each other and self-assemble into a layered organisation providing increased uniformity in self-assembly compared to non-lipidated β^3^-peptides.

This strategy has also been utilised by many groups for the assembly of α-peptides containing β-sheet forming sequences and has allowed the generation of a number of materials for various applications and is expertly reviewed by Stupp and colleagues.^10^

**Fig. 1:**
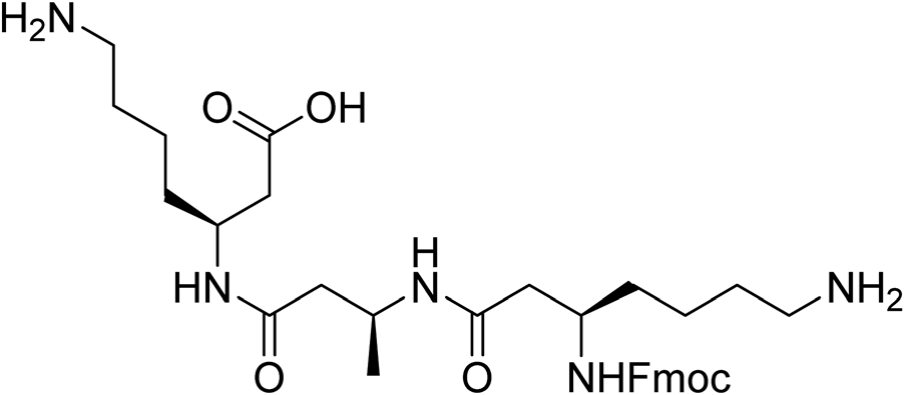
Structure of Fmoc-β-(KAK).

However, the synthesis and purification of lipidated peptides can be problematic due to the overall hydrophobicity of the peptides as well as the propensity for these peptides to assemble during synthesis and purification. Therefore, to explore the effect of other N-terminal capping on the self-assembly, fluorenylmethoxycarbonyl (Fmoc) group onto the N-terminus in place of the acyl chain. Fmoc has been used extensively by a number of groups to induce self-assembly in α-peptides and create a range of materials including hydrogels and scaffolds, which have been utilised for a number of *in vivo* applications.^11–18^ The Fmoc-protected α-peptides are able to stack atop each other to initiate the self-assembly to form fibrillar structures.^11,12^ We also anticipated that the carbamate present in the Fmoc-protected β-peptide structure, depicted in Fig. 1, would satisfy the hydrogen bonding requirements for the self-assembly of β-peptides.^2,6^

Herein, we describe the modification of an acylated β^3^-peptide with sequence Lys-Ala-Lys-OH, which has previously been shown to produce fibrillar structures.^8^ We substituted the acyl-chain at the N-terminus for an Fmoc group for ease of synthesis and purification. Unexpectedly, we observed the formation of regular spherical particles as opposed to the fibrillar structures commonly associated with β^3^-peptide assemblies. These spherical particles were found to be stable over a range of temperatures and able to be stored for prolonged periods. In addition, we were able to encapsulate these particles with cargo that could also be delivered to cells. This is the first description of sub-200 nm particles from the assembly of β^3^-peptides.

## Materials and methods

### Preparation of particles

β^3^-tripeptides composed of β-lysine–β-alanine–β-lysine residues coupled with a fluorenylmethoxycarbonyl (Fmoc) protecting group {Fmoc-β-(KAK)} were synthesized by Fmoc solid phase peptide synthesis and purified to 98% purity by HPLC-MS. Briefly, Preparative High-Performance Liquid Chromatography (HPLC) was performed using an Agilent 1200 series HPLC system fitted with a reverse-phase preparative (C18, 300 Å, 5 μm, 10 mm × 250 mm) column. Analytical HPLC was performed using an Agilent 1100 series HPLC system fitted with a reverse-phase analytical (C18, 300 Å, 5 μm, 4.6 mm × 150 mm) column. Mass spectra were acquired to confirm peptide identity using an Agilent 1100 MSD SL ion trap mass spectrometer (Agilent Technologies, Santa Clara, California, USA).

Fmoc-β-(KAK) peptides were prepared by dissolving 5 mg of peptide with 1.64 mL of sterilized water at room temperature (RT) to prepare a stock concentration of 5 mM. 100 μL aliquots (5 mM) were diluted with 400 μL sterilized water to prepare 1 mM samples. Samples underwent bath sonication using the Ultrasonic Cleaner (General Vet Products, Fairy Meadow, NSW, Australia) for an initial 10 minutes prior to undergoing further characterisation.

### Particle size determination

Particle size was determined through use of DLS and NTA. Particles were suspended in sterilized water and analysed at room temperature by DLS (ZetaSizer Nano ZS, Malvern Instruments, Worcestershire, UK). Mean diameter, size distribution and polydispersity index (PDI) were measured. Particle size and distribution was also determined using the Zetaview® PMX-120 Nanoparticle Tracking Analyzer (NTA) (laser = 488 nm, sensitivity = 80 AU, shutter speed = 100 s^−1^, frame rate = 30 s^−1^). Samples were injected using a 1 : 10 dilution of Fmoc-β-(KAK) stock (1 mM).

### Atomic force microscopy (AFM)

AFM was used to verify results obtained from DLS for size and shape of empty and encapsulated particles. Briefly, 10 μL of particles suspended in sterilized water was placed on a sterilized, glass slide. Samples were then imaged with a FastScan AFM using a Scanasyst-Fluid+ probe in ScanAsyst Fluid mode at 1 Hz. Scan sizes were 5 μm or 1 μm squares. Images were processed using a sequence of plane fitting and offset flattening using Gwyddion 2.29 (Gwyddion.net) software.

### Transmission electron microscopy (TEM)

To visualize the shape and size of the peptide particles, peptide samples dissolved in water were imaged by TEM. A 5 μL of peptide sample was applied to the surface of glow-discharged and negatively charged continuous carbon grids and stained with 2% uranyl acetate solution. The excess stain was removed using filter paper and grids were air-dried. All samples were imaged on an FEI Tecnai F30 electron microscope equipped HAADF STEM detector and a Gatan quantum 965 energy filter and an upper CETA 4x4k CMOS camera, under a working voltage of 200 kV at the Ian Homes Imaging Center at Bio21 Institute of the University of Melbourne. The samples were imaged primarily at ×59 000 and respective scale bars are denoted in the TEM images.

### Stability of Fmoc-β-(KAK) particles

To assess the stability of particles, 500 μL aqueous samples at 1 mM concentrations were placed in room temperature, 4, −20 and −80 °C environments, avoiding light sources. Size distribution was determined with DLS at day 0, then again at day 7 and 14. Samples stored below 0 °C were frozen and thawed at day 0 to assess the impact on particle size.

### Encapsulation of a fluorophore

From the 5 mM stock solution, 100 μL aliquots of Fmoc-β-(KAK) were taken and combined with 25 μL aliquots of Quasar (LGC BioSearch) from 100 μg mL^−1^ stock solution and 375 μL of sterilized water to make up 500 μL samples of 1 mM Fmoc-β-(KAK) + 5 μg Quasar. Control samples of Quasar alone was also made up with 25 μL of Quasar stock solution in 475 μL sterilized water. Fmoc-β-(KAK) + Quasar and control samples were sonicated for 10 minutes using the same conditions as described above. Samples were then centrifuged at 20 000 rpm for 10 minutes to separate the Quasar-encapsulated Fmoc-β-(KAK) particles from free Quasar in treatment samples. 300 μL of supernatant was pipetted into fresh microcentrifuge tubes and analysed using microplate reader (VersaMax™) and NanoDrop (Thermo Scientific™) to measure absorbance at 644 nm of free, unencapsulated Quasar. Analysis was conducted, using unpaired *t*-tests to identify statistical significance between samples.

### Neuro-2A cell cytotoxicity tests

Neuro 2A cells (passage 7) were transferred to a 96 well plate previously coated with poly-l-lysine in a density of 20 000 cells per well and 100 μL of DMEM media + 10% FBS + 1% penicillin and left to settle for 24 hours. Particles were added to cells in doses of 0.2, 2, 10, 20, 60 and 120 μg and left for 24 hours. Cell viability was determined using MTS assay, using MTS reagent and DMEM solution in a ratio of 1 : 6 in 100 μL. Assay was incubated for 35 minutes prior to measuring absorbance at 490 nm by microplate reader.

### Cellular uptake of fluorescent particles

Cells were cultured using the same method as stated above, and were transferred to a 48 well plate in a density of 25 000 cells per well. Quasar-encapsulated Fmoc-β-(KAK) particles or Quasar alone was added to cells at 12.5 and 25 μg doses and returned to the cell culture incubator for 24 hours. Following incubation, the cells were then fixed using 4% paraformaldehyde (PFA) before investigating cellular uptake using fluorescence microscopy.

## Results and discussion

### Characterisation of self-assembled Fmoc-β-(KAK) particles

**Fig. 2:**
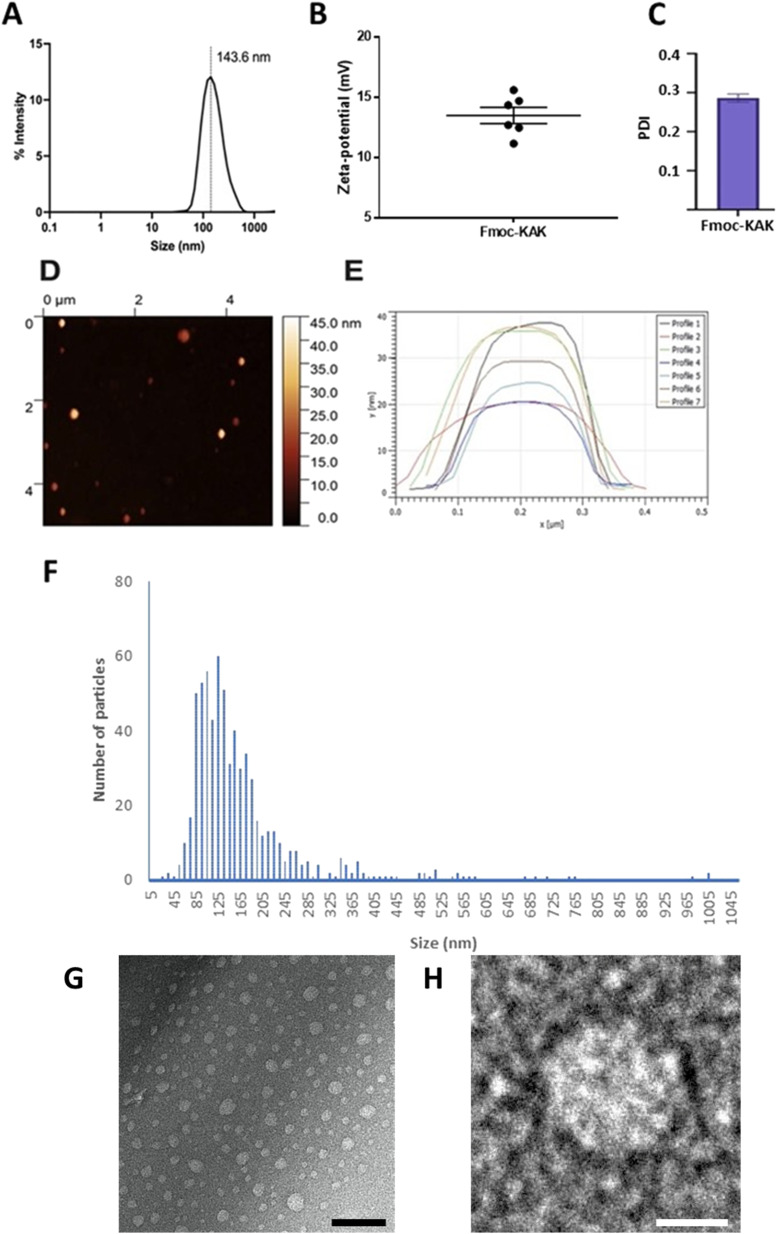
(A) Mean size distribution of Fmoc-β-(KAK) particles analysed using Dynamic Light Scattering (DLS). The *z*-average is reported and depicted by the vertical black dotted line. (B) Zeta-potential as reported by DLS. (C) Bar graph showing the polydispersity indexes of particles measured by DLS. Data is presented as the mean ± SEM atomic force microscopy (AFM) image of (D) Fmoc-β-(KAK) particles in water and (E) line profiles measuring individual particle diameters. (F) Distribution of the number and size of particles as determined by NTA. *n* = 4–9. (G) TEM image of Fmoc-β-(KAK) particles (scale bar = 200 nm) and (H) an image of a single particle (scale bar = 50 nm).

Fmoc-β-(KAK) particles were suspended in sterilised milliQ water and underwent DLS analysis to determine average size distributions of particles and their zeta-potential. The mean size for particles generated by Fmoc-β-(KAK) self-assembly was found to be 143 nm as observed by DLS (Fig. 2A). The homogeneity of these particles was confirmed by a relatively low polydispersity index (PDI) of 0.286. The level of variability, as denoted by the low PDI figure, is typically considered to fall within the ideal range for pharmaceutical applications.^19^ Furthermore, the zeta-potential of these particles was measured at +13 indicating these particles to be mildly cationic and may also indicate that the lysine residues within the sequence are solvent exposed (Fig. 2B and C). Atomic force microscopy, nanoparticle tracking analysis and transmission electron microscopy all confirmed the presence of particles consistent with the findings by DLS (Fig. 2D–H). Previous literature reports nanoparticles derived from the assembly of cell penetrating peptides (CPPs) to be larger than the Fmoc-β-(KAK) particles, with diameters ranging between 230 and 500 nm.^20^ In addition, peptides comprised of cyclic β-amino acids were found to self-assemble into vesicle-like structures.^21^ Furthermore, larger co-block polypeptide-based particles containing Lys_200_-β-Gly_50_ are found to average ∼350 nm when generated using a similar protocol to the current study.^22^

### Storage of Fmoc-β-(KAK) particles

**Fig. 3:**
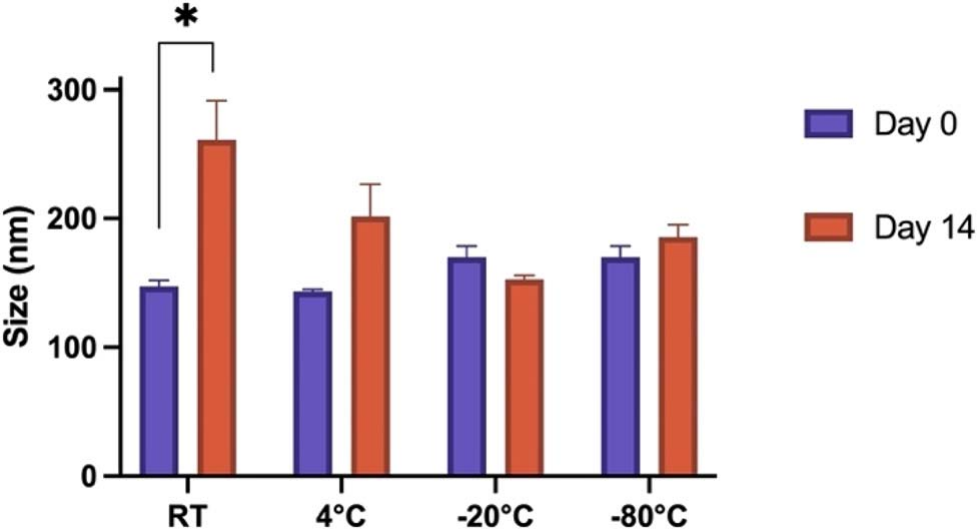
Grouped bar graphs depicting the difference in particle diameter following 14-day storage of Fmoc-β-(KAK) particles in different temperature environments. Data is presented as mean ± SEM and unpaired *t*-tests were conducted between individual groups to determine if significant changes in size occur following storage of particles. *n* = 3–6.

Various storage methods were evaluated to determine stability and applicability of Fmoc-β-(KAK) particles. Particle size was determined on day 0 by DLS following synthesis and then again after 14 days of storage in various conditions. *t*-Tests were conducted on individual groups to determine significance. When left at RT for 14 days, Fmoc-β-(KAK) particles significantly increased in size from 147 nm to 261 nm (Fig. 3). Other storage temperatures were not found to have any significant effect on particle size, although the storage of particles at 4 °C appeared to demonstrate a trend for particle size to increase, albeit modestly from 143 to 201 nm after 14 days.

Freezing Fmoc-β-(KAK) particles at both −20 °C and −80 °C resulted in very small changes in particle size, where −80 °C slightly increased from 169 nm to 185 nm after 14 days and storage at −20 °C marginally decreased from 169 nm to 153 nm in diameter (Fig. 3). Fig. 3 suggests that freezing Fmoc-β-(KAK) particles is the best method of short-term storage. There is significant conjecture surrounding the appropriate storage conditions for nanoparticles. For lipid-based nanoparticles (LNPs), there are contrasting opinions on the best temperatures at which to store particles in order to reduce aggregation and disintegration.^23^ One previous study showed that refrigeration of LNPs caused an increase in size of particles from 150 nm to 190 nm in diameter.^24^ However, other literature contradicts these findings and claim that storage of LNPs at 4 °C kept particle size stable over the long term, and that freezing at −20 °C resulted in increased aggregation and particle sizes.^24^ However, the current study suggests that Fmoc-β-(KAK) particles should be frozen, ideally at −20 °C to maintain stability.

### Fmoc-β-(KAK) particles can encapsulate hydrophobic cargo

**Fig. 4:**
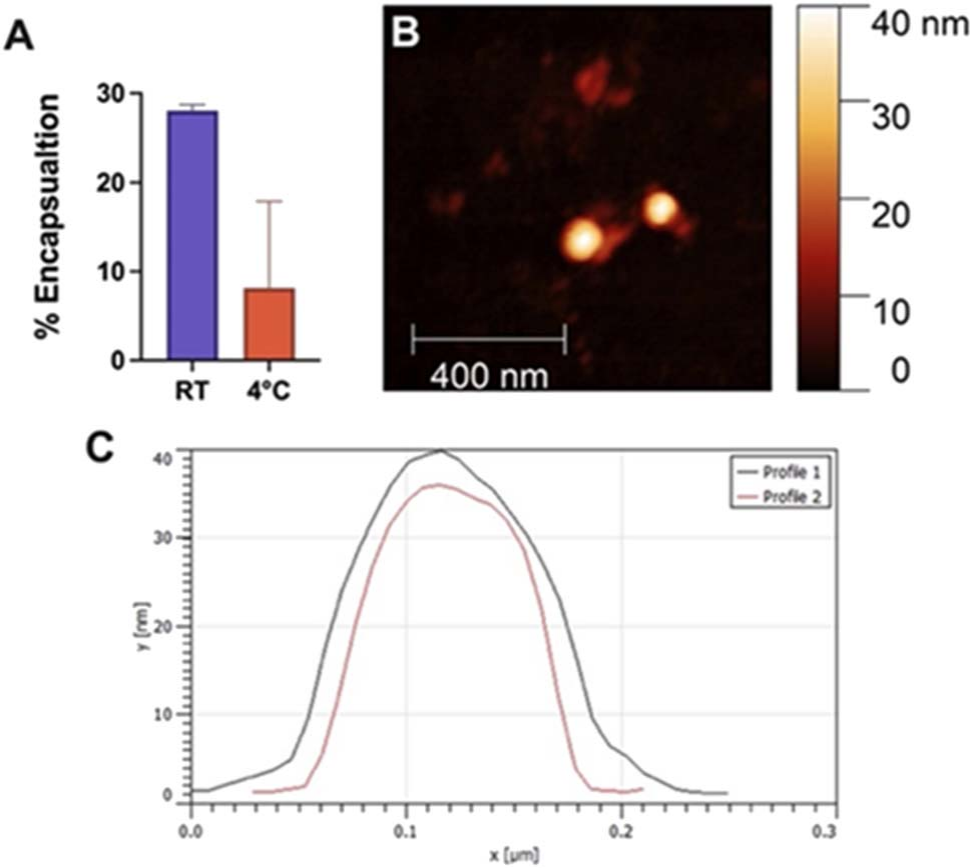
Bar graph depicting the (A) percentage of Quasar670 encapsulation by Fmoc-β-(KAK) at either RT or 4 °C. Data is presented as mean ± SEM, *n* = 3–5. (B) Atomic force microscopy identifying individual particles following encapsulation of Quasar670 at RT. (C) Line profiles of particles demonstrate approximate diameters.

Encapsulation efficiency was investigated by sonicating Quasar670, a fluorescent dye, with Fmoc-β-(KAK) particles and measuring absorbance levels of free, un-encapsulated Quasar670 following centrifugation. The percentage of Quasar670 encapsulated by Fmoc-β-(KAK) particles was calculated from control samples of Quasar670 alone. Quasar670 encapsulation by Fmoc-β-(KAK) was more effective when performed at RT environments compared to preparation at 4 °C. The addition of Quasar670 to the particle synthesis at RT yielded an average 28.0% of encapsulated Quasar670, whereas only 8.1% of Quasar670 was encapsulated when added to Fmoc-β-(KAK) at 4 °C (Fig. 4A). The encapsulation efficiency of Fmoc-β-(KAK) particles at RT is similar to other liposomal and PEGylated micelle nanoparticles.^25,26^ However, while such nanoparticles are designed to maximise encapsulation efficiency, the Fmoc-β-(KAK) particles have not been modified for this purpose, yet achieved a level of encapsulation. In addition, the lysine residues on the particles' surface provides great potential for future tailoring, to enhance encapsulation efficiency.

Fmoc-β-(KAK) particles encapsulated with Quasar670 at RT were imaged using AFM to determine changes in particle size. DLS was unable to be conducted due to the inherent fluorescence of Quasar670 interrupting the DLS signalling. Analysis of AFM images confirmed the particles to have similar diameters as those described in Fig. 1. Previous research surrounding peptide-based particles have found similar results, where CPPs complexed with siRNA were found to range between 120 and 160 nm in size.^23^ Furthermore, another self-assembling peptide, RICK-siRNA particles assembled in water were found to have a mean diamater of 300 nm, with a range of 150–500 nm.^27^ We attempted encapsulation of nucleic acids into Fmoc-β-(KAK) particles and found that large complexes formed with sizes ranging from 200–2000 nm (Fig. S4†).

Given the likely positive charge on the exterior of the particle, it is hypothesised that the highly negatively charged backbone of the nucleic acids bind to the corona of the particle and subsequent base-pairing drives the formation of macromolecular assemblies.

### Cellular biocompatibility of Fmoc-β-(KAK) particles and delivery of cargo to cells

**Fig. 5:**
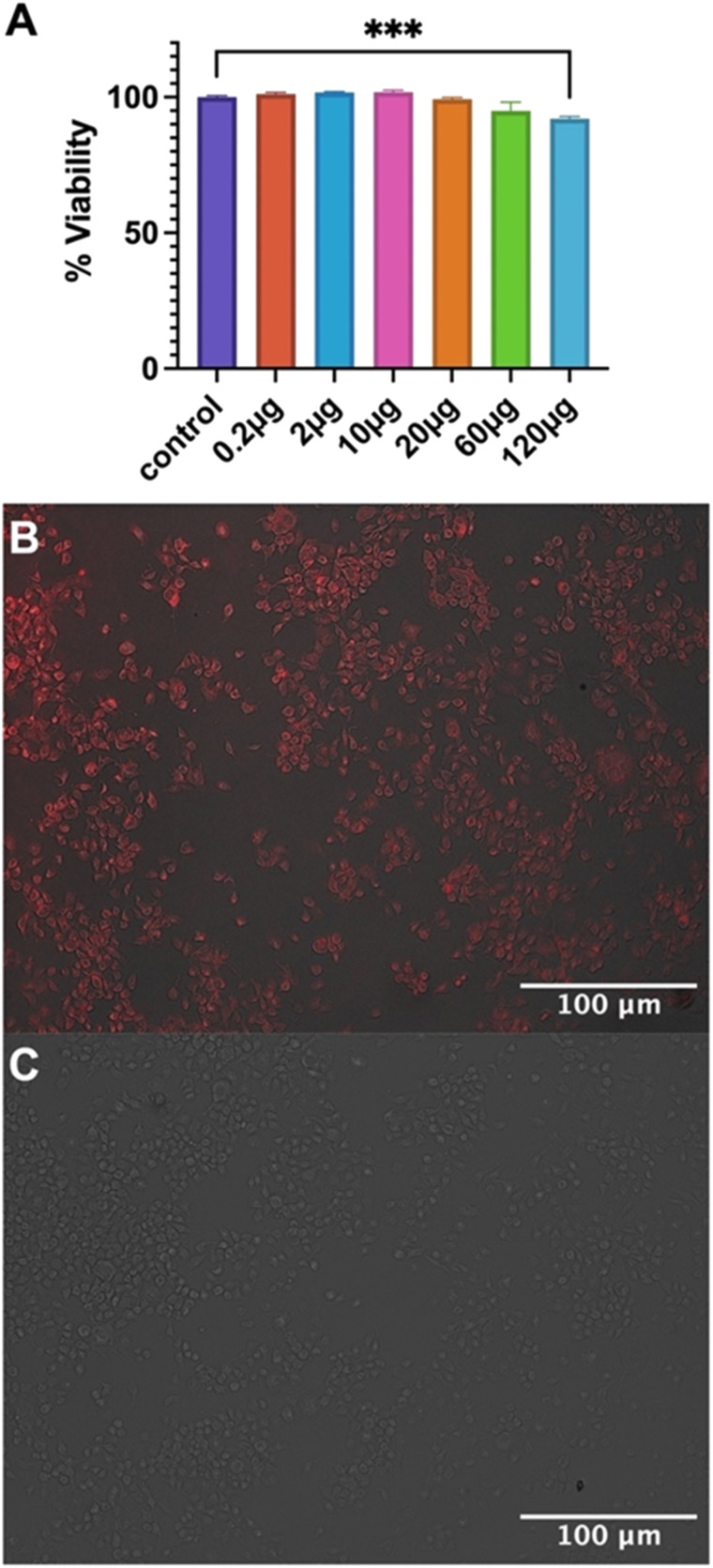
Bar graph highlighting (A) cellular toxicity of Fmoc-β-(KAK) particles after incubation of increasing doses in Neuro 2A cells for 24 hours. Data is presented as the average percentage cell viability ± SEM with one-way ANOVA performed to determine significance. Fluorescent images highlighting (B) cellular internalisation of 100 μg Quasar670-encapsulated Fmoc-β-(KAK) particles after 24-hour incubation in Neuro 2A cells and (C) control image of Neuro 2A cells treated without particles at 100× magnification. *n* = 3–6.

Biocompatibility of empty Fmoc-β-(KAK) particles was measured using MTS assays on a neuroblastoma cell line and the percentage of cell viability was calculated. When compared to the control group, 0.2, 2 and 10 μg doses were not toxic to cells, averaging 101.2%, 101.7% and 101.8% viability respectively (Fig. 5A). 20 μg and 60 μg treatment groups reported a slight reduction in viability (99.3% and 95.0% respectively), though neither treatment groups were found to be significant. Finally, significance was only found in the 120 μg treatment group, where a reduction in viability of 92.1% was found (Fig. 5A). These findings are similar to previous literature, where particles derived from CPPs were not found to elicit any cytotoxicity with 100 μM of peptide complexed with siRNA.^23^ However, it should be noted that the concentrations used in the present study were substantially higher. Furthermore, studies have also shown that peptide particles delivering siRNAs at higher concentrations resulted in a decrease in cell viability of 20%, although it is not clear what concentration of peptide was used to administer the siRNA.^27^

Nevertheless, the current study shows that despite the inherent cationic charge of Fmoc-β-(KAK), minimal toxicity was observed at high doses.

Cellular uptake assays were also performed to determine the ability of Fmoc-β-(KAK) particles to enter cells. Quasar670-encapsulated Fmoc-β-(KAK) particles were administered to a neuroblastoma cell line and incubated for 24 hours before uptake was investigated using fluorescence microscopy. Images at 100× magnification show Quasar fluorescence indicating that Fmoc-β-(KAK) particles had successfully internalised into cells following 24 hours incubation, whilst control cells to which Quasar alone delivered, failed to indicate any fluorescence (Fig. 5B and C) as observed for other studies using peptide-based particles *in vitro*.^23,27^

## Conclusions

Previously, we have described a number of fibrous materials derived from the self-assembly of β^3^-peptides that have been modified with N-terminal acylation. In order to explore the impact of other N-capping methods, we utilised the Fmoc-group to facilitate self-assembly. We were able to observe the formation of regular-sized nanoparticles from aqueous solvents that proved to be stable in a range of refrigerated conditions. These particles were able to encapsulate hydrophobic cargo and deliver this cargo to cells with limited cytotoxicity. This is the first description of β^3^-peptide-derived nanoparticles with potential for pharmaceutical applications.

## Author contributions

The manuscript was written through contributions of all authors. All authors have given approval to the final version of the manuscript.

## Conflicts of interest

There are no conflicts of interest to declare.

## Supplementary Material

RA-013-D3RA04514E-s001
