# Supplementary material for: A switch in N-terminal capping of β-peptides creates novel self-assembled nanoparticles
Source: RSC Adv. 2023 Oct 9;13(42):29401–7. doi: 10.1039/d3ra04514e (PMC10561372; doi:10.1039/d3ra04514e)
Supplement: RA-013-D3RA04514E-s001 [file RA-013-D3RA04514E-s001.pdf]

Electronic Supplementary Material (ESI) for RSC Advances.
This journal is © The Royal Society of Chemistry 2023

Supporting Information for;

A switch in N-terminal capping of beta-peptides creates novel self-assembled nanoparticles

Yi-Kai Chen,[#a,c] *Isabella A. Simon,* [#a,c] *Ivan Maslov,* [a,c] *Ivan E. Oyarce-Pino,* [a,c] *Ketav Kulkarni,* [b,c] *Denham Hopper,* [a,c] *Marie-Isabel Aguilar,*[b,c] *Naveen Vankadari,*[d] *Brad Broughton* [a,c] *and Mark P Del Borgo* [a,c*]

Methods and Materials:

Peptide synthesis

All coupling reagents and beta amino acids were purchased from GL Biochem (Shanghai, China). β^3-tripeptides composed of β-lysine-β-alanine-β-lysine coupled with a fluorenylmethoxycarbonyl (Fmoc) protecting group were synthesised by Fmoc solid phase peptide synthesis and purified to 98% purity by HPLC-MS. The peptide was synthesized on a 0.1 mmol scale using standard Fmoc chemistry on Wang resin (0.1 mmol/g loading). The resin was swollen in DMF (4 mL) and then soaked overnight with gentle agitation with Fmoc-protected β amino acid (2.1 eq. to resin loading), HBTU (2 eq. to resin loading), HOBt (2 eq. to resin loading), DMAP (10 mol%) and DIPEA (3 eq. to resin loading), which was all dissolved in DMF (4 mL). Next, the resin was thoroughly washed with DMF (3 × 4 mL) and the Fmoc protecting group on the amino acid was removed by soaking the resin twice in 20% piperidine, with 0.1 M HOBt, in DMF (4 mL) for 15 min each. The resin was washed with DMF (3 × 5 mL), soaked in Fmoc-protected amino acid (2.1 eq. to resin loading), dissolved in DMF (4 mL) along with HBTU (2 eq. to resin loading), HOBt (2 eq. to resin loading) and DIPEA (3 eq. to resin loading), for 2 h. β Peptide elongation cycle was then repeated until the sequence was complete. The final Fmoc-protecting group was left on the peptide chain. The resin was then washed with DMF (2 × 4 mL), CH2Cl2 (2 × 4 mL), Et2O (2 × 4 mL), air dried for 10 min, and transferred to a 15 mL vial for cleavage.

Cleavage was performed on the resin (0.1 mmol), by treating the resin with a cleavage solution (10 mL) comprising of H2O (5% v/v) in Trifluoroacetic acid (TFA), for 90 minutes. TFA was then evaporated under a stream of N2 and the peptide was precipitated by addition of Et2O (50 mL). The precipitate was filtered and redissolved in 50% aqueous CH3CN for lyophilisation.

Peptide Purification

Preparative High-Performance Liquid Chromatography (HPLC) was performed using an Agilent 1200 series HPLC system fitted with a reverse-phase preparative (C18, 300 Å, 5 µm, 10 mm × 250 mm) column. Analytical HPLC was performed using an Agilent 1100 series HPLC system fitted with a reverse-phase analytical (C18, 300 Å, 5 µm, 4.6 mm × 150 mm) column. Mass spectra were acquired to confirm peptide identity using an Agilent 1100 MSD SL ion trap mass spectrometer (Agilent Technologies, Santa Clara, California, USA).

Preparation of particles

Fmoc-β-KAK peptides were prepared by dissolving 5mg of peptide with 1.64mL of sterilised water at room temperature (RT) to prepare a stock concentration of 5 mM. 100 µL aliquots (5 mM) were diluted with 400 µL sterilised water to prepare 1 mM samples. Samples underwent bath sonication using the Ultrasonic Cleaner (General Vet Products, Fairy Meadow, NSW, Australia) for an initial 10 minutes prior to undergoing further characterisation.

Fmoc-β-(KAK)-OH

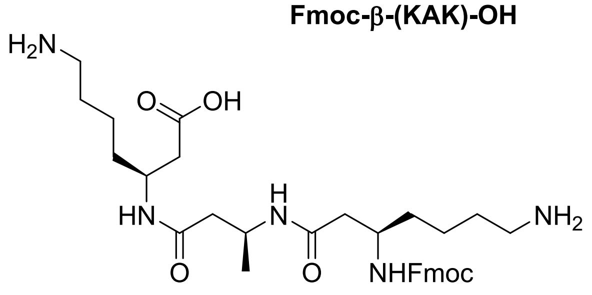

Chemical Formula: $C_{33}H_{47}N_5O_6$
Exact mass: 611.6
m/z $C_{33}H_{49}N_5O_6^{2+}$: 306.0

MS Fmoc–β–KAK-OH

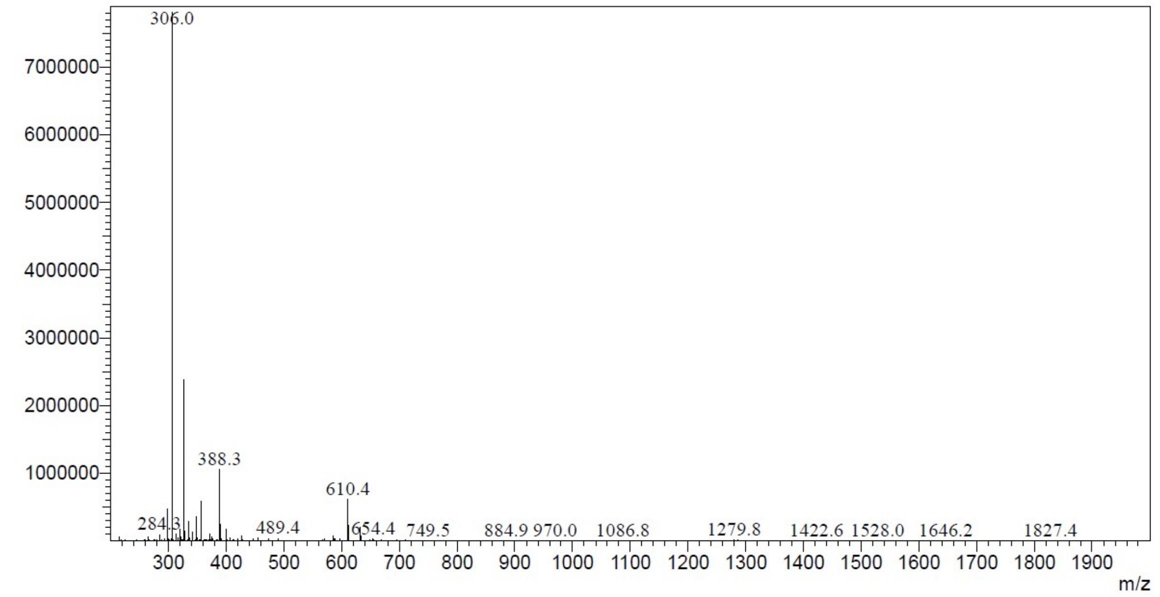

LC Fmoc–β–KAK-OH

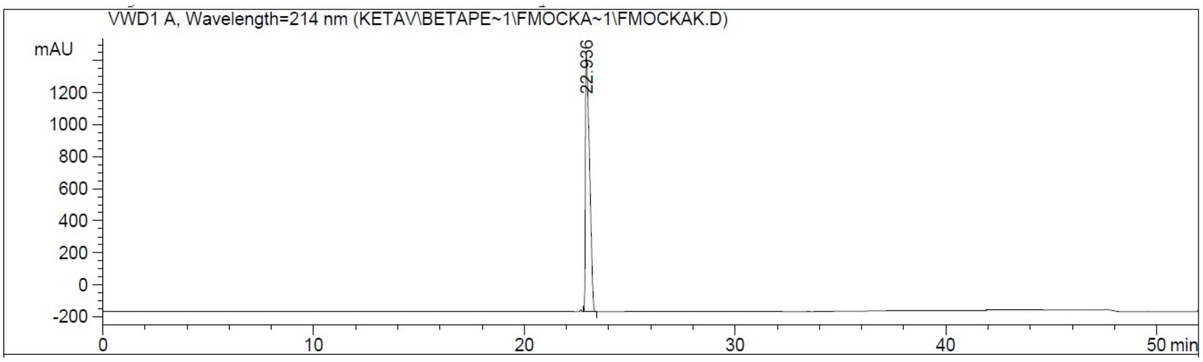

Figure S1: HPLC and MS of purified peptide

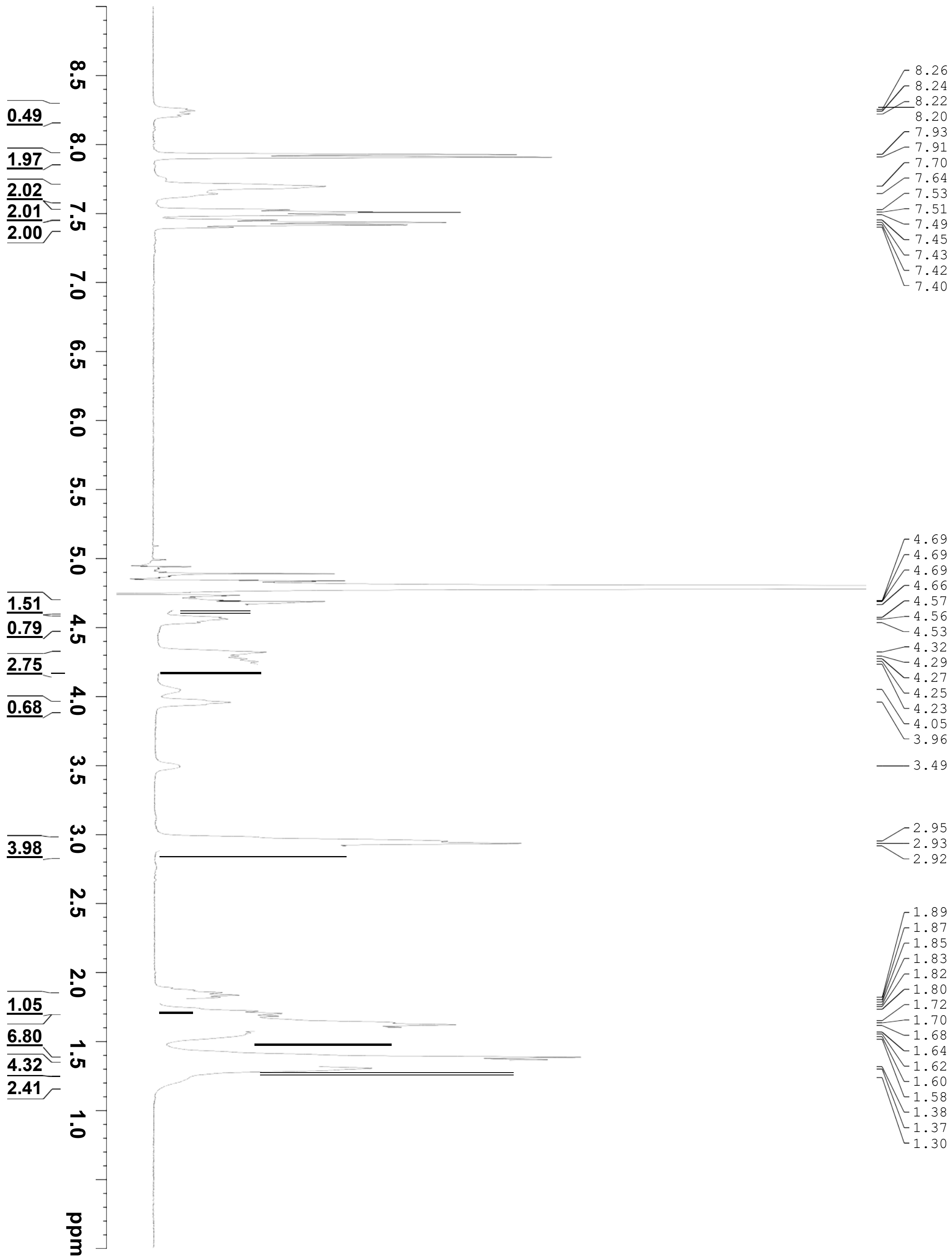

Figure S2: NMR spectra of Fmoc-β-KAK

Particle Size Determination

Particle size was determined through use of DLS and NTA. Particles were suspended in sterilised water and analysed at room temperature by DLS (ZetaSizer Nano ZS, Malvern Instruments, Worcestershire, UK). Mean diameter, size distribution and polydispersity index (PDI) were measured. Particle size and distribution was also determined using the ZetaView® PMX-120 Nanoparticle Tracking Analyzer (NTA) (Laser = 488nm, Sensitivity = 80 AU, Shutter Speed = 100s -1, Frame Rate = 30/s). Samples were injected using a 1:10 dilution of Fmoc–β–KAK stock (1 mM), (Max Area = 1000 px, Min Area = 10 px, Max Brightness = 30)

Atomic Force Microscopy (AFM)

AFM was used to verify results obtained from DLS for size and shape of empty and encapsulated particles. Briefly, 10 µL of particles suspended in sterilised water was placed on a sterilised, glass slide. Samples were then imaged with a FastScan AFM using a Scanasyst-Fluid+ probe in ScanAsyst Fluid mode at 1 Hz. Scan sizes were 5 µm or 1 µm squares. Images were processed using a sequence of plane fitting and offset flattening using Gwyddion 2.29 (Gwyddion.net) software.

Stability of Fmoc–β–KAK particles

To assess the stability of particles, 500 µL aqueous samples at 1 mM concentrations were placed in room temperature, 4, -20 and -80°C environments, avoiding light sources. Size distribution was determined with DLS at day 0, then again at day 7 and 14. Samples stored below 0°C were frozen and thawed at day 0 to assess the impact on particle size.

Encapsulation of Quasar

From the 5 mM stock solution, 100 µL aliquots of Fmoc–β–KAK were taken and combined with 25 µL aliquots of Quasar (LGC BioSearch, Extinction coefficient at Lambda max:250000) from 100 µg/mL stock solution and 375 µL of sterilised water to make up 500 µL samples of 1 mM Fmoc–β–KAK + 5µg Quasar. Control samples of Quasar alone was also made up with 25 µL of Quasar stock solution in 475 µL sterilised water. Fmoc–β–KAK + Quasar and control samples were sonicated for 10 minutes using the same conditions as described above. Samples were then centrifuged at 20,000rpm for 10 minutes to separate the Quasar-encapsulated Fmoc–β–KAK particles from free Quasar in treatment samples. 300 µL of supernatant was pipetted into fresh microcentrifuge tubes and analysed using microplate reader (VersaMax™) and NanoDrop (Thermo Scientific™) to measure absorbance at 644 nm of free, unencapsulated Quasar. Analysis was conducted, using unpaired t-tests to identify statistical significance between samples.

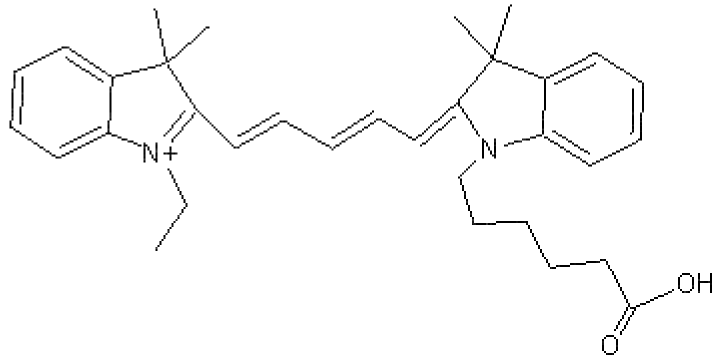

Figure S3: Structure of Quasar 670 used in the encapsulation study.

Single-stranded DNA (4 µM, (5'-ACCATCGACCGTTGATTGTACC-3, 6749.36 Da, Micromon platform, Monash University) was also combined with Fmoc–β–KAK and followed the above protocol to assess % encapsulation. DLS analysis of Fmoc–β–KAK particles with ssDNA demonstrates many sizes and poor polydispersity (Fig S3)

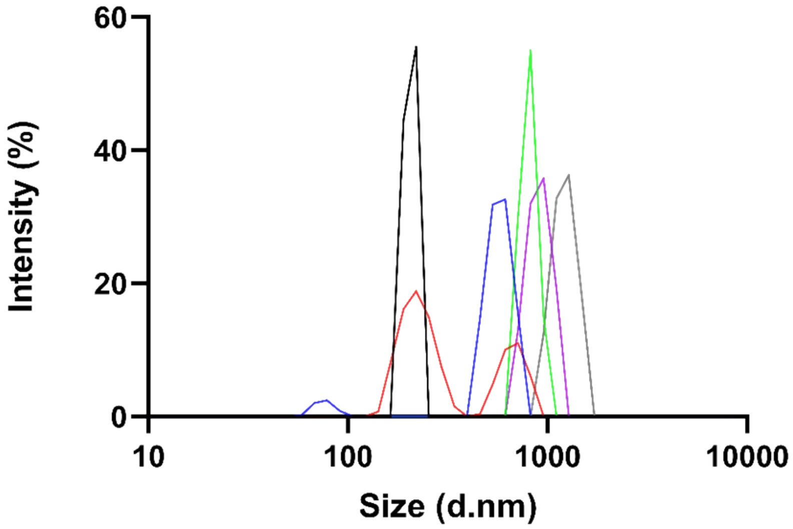

Figure S4: DLS size distribution of Fmoc–β–KAK complexed with 4 uM of ssDNA (n = 6 batches)

Neuro-2A Cell Cytotoxicity Tests

Passage 7 of Neuro 2a cell line was transferred to a 96 well plate previously coated with poly-L-lysine in a density of 20,000 cells/well and 100 µL of DMEM media + 10% FBS +1% penicillin and left to settle for 24 hours. Particles were added to cells in doses of 0.2, 2, 10, 20, 60 and 120 µg and left for 24 hours. Cell viability was determined using MTS assay, using MTS reagent and DMEM solution in a ratio of 1:6 in 100 µL as previously described. [S1] Assay was incubated for 35 minutes prior to measuring absorbance at 490 nm by microplate reader.

Cellular Uptake of fluorescent particles

Cells were cultured using the same method as stated above, and were transferred to a 48 well plate in a density of 25,000 cells/well. Quasar-encapsulated Fmoc–β–KAK particles or Quasar alone was added to cells at 12.5 and 25 µg doses and returned to the cell culture incubator for 24 hours. Following incubation, the cells were washed with PBS, then fixed using 4% paraformaldehyde (PFA) before investigating cellular uptake using fluorescence microscopy.

References

S1: Mosmann T. 1983. Rapid colorimetric assay for cellular growth and survival: Application to proliferation and cytotoxicity assays. Journal of Immunological Methods. 65(1-2):55-63. https://doi.org/10.1016/0022-1759(83)90303-4
